# On Defining and Finding Islands of Trees and Mitigating Large Island Bias

**DOI:** 10.1093/sysbio/syab015

**Published:** 2021-03-22

**Authors:** Ana Serra Silva, Mark Wilkinson

**Affiliations:** 1 Department of Life Sciences, The Natural History Museum, London SW7 5BD, UK; 2 School of Earth Sciences, University of Bristol, Bristol BS8 1RL, UK

## Abstract

How best can we summarize sets of phylogenetic trees? Systematists have relied heavily on consensus methods, but if tree distributions can be partitioned into distinct subsets, it may be helpful to provide separate summaries of these rather than relying entirely upon a single consensus tree. How sets of trees can most helpfully be partitioned and represented leads to many open questions, but one natural partitioning is provided by the islands of trees found during tree searches. Islands that are of dissimilar size have been shown to yield majority-rule consensus trees dominated by the largest sets We illustrate this large island bias and approaches that mitigate its impact by revisiting a recent analysis of phylogenetic relationships of living and fossil amphibians. We introduce a revised definition of tree islands based on any tree-to-tree pairwise distance metric that usefully extends the notion to any set or multiset of trees, as might be produced by, for example, Bayesian or bootstrap methods, and that facilitates finding tree islands *a posteriori*. We extract islands from a tree distribution obtained in a Bayesian analysis of the amphibian data to investigate their impact in that context, and we compare the partitioning produced by tree islands with those resulting from some alternative approaches. Distinct subsets of trees, such as tree islands, should be of interest because of what they may reveal about evolution and/or our attempts to understand it, and are an important, sometimes overlooked, consideration when building and interpreting consensus trees. [Amphibia; Bayesian inference; consensus; parsimony; partitions; phylogeny; Chinlestegophis.]

Phylogenetic analyses may recover multiple trees, either by design (e.g., Bayesian inference and resampling techniques) or because the data support multiple sufficiently optimal solutions. Typically, in such cases, a consensus tree is used to provide a graphical summary of the multiple trees. There are many consensus methods, but the strict (Sokal and Rohlf 1981) and the majority-rule (Margush and McMorris 1981) are among the most commonly used, and easiest to interpret. Majority-rule consensus trees display only those splits present in a majority of the input trees, and, decorated with the frequencies of occurrence of the displayed splits, are routinely used to summarize bootstrap and Bayesian analyses. Strict consensus trees display just those splits that are present in all input trees, a subset of those displayed by majority-rule trees, and are mainly used to summarize sets of most parsimonious trees (MPTs). Despite concerns that summarizing MPTs with the majority-rule consensus is potentially misleading (e.g., Wilkinson and Benton 1996; Sharkey and Leathers 2001; Sumrall et al. 2001; Sharkey et al. 2013), some workers still use the majority-rule method as if it were unproblematic (e.g., Taylor et al. 2008; Coiffard et al. 2013; Gunnell et al. 2017; Pardo et al. 2017).

It has long been appreciated that sets of inferred trees may comprise (may be partitioned into) distinct subsets (families or islands) of trees, which it may be useful to summarize separately (Hendy et al. 1988; Maddison 1991). These subsets have mostly been defined based on tree-to-tree distances (e.g., families of trees, Hendy et al. 1988), including those based on branch rearrangement metrics (e.g., tree islands, Maddison 1991). As originally defined, islands are sets of trees such that any pair is connected by a series of included trees, each of which is sufficiently similar to the adjacent members of the series. Islands were discovered through application of heuristic branch swapping to different starting trees. Initially, the main concerns were that different islands could have major implications for character evolution (Maddison 1991) and that heuristic searches could get trapped in suboptimal islands (e.g., Olmstead et al. 1993; Olmstead and Palmer 1994), so that large numbers of starting trees should be used to improve the chances of finding all islands. In a parsimony context, it has been shown that if most of the overall variation in tree topology is between islands of trees and the islands contain very disparate numbers of trees, then the majority-rule consensus will be dominated by the largest islands. The concern is that such “large island bias” will conceal important variation in tree topology (Sumrall et al. 2001). In the extreme, if the size of one tree subset sufficiently outnumbers all others, then the majority-rule consensus will show only those relationships found in the largest island, thus losing the information in smaller islands. Beyond parsimony, the number of such subsets, the distances between them, and their posterior probabilities can affect chain convergence in Bayesian analyses (Höhna and Drummond 2011; Lakner et al. 2008), and the presence of multiple sets of equally optimal trees (terraces) can negatively affect tree search in maximum likelihood analyses of concatenated alignments (Sanderson et al. 2011; Sanderson et al. 2015). However, in model-based phylogenetics, subsets of trees are seldom explored outside of the tree search context, and it is thus unknown how prevalent the issue of large island bias is when summarizing tree distributions obtained by Bayesian and likelihood analyses.

Here, we revisit the problem of large island bias, illustrate it with a recent empirical example, investigate its cause in this case, and consider how it may be mitigated. We briefly review the use of tree-to-tree distance metrics in defining subsets of trees. We extend the concept of islands of trees to encompass multisets (weighted sets) of trees, as may result from resampling methods and Bayesian analyses, and to allow them to be based on any tree-to-tree distance. We consider how islands can be discovered *a posteriori* and identify islands in a tree distribution recovered by Bayesian inference. We compare islands and some alternative approaches to partitioning these empirical sets of trees. We seek to highlight the potential importance of subsets of trees, such as islands, and motivate further work on their discovery and interpretation.

## DEFINING ISLANDS OF TREES

The existence of distinct subsets of similar trees and implications for consensus were first considered by Hendy et al. (1988). They conceived of families of trees as subsets such that all members are only a small distance from all other members, defined a family more formally as all trees within a fixed distance from a tree *T*, and employed clustering based on a pairwise tree-to-tree distance to identify families in their examples. Their definition and clustering used the symmetric difference on full splits (Hendy et al. 1984), also known as the partition metric or Robinson–Foulds distance (RF, Robinson and Foulds 1981), as the tree-to-tree distance. Several similar heuristic clusterings of trees have been developed subsequently (e.g., Stockham et al. 2002; Guénoche 2013). Somewhat differently, Maddison (1991) defined the mutually exclusive subsets he denoted as tree islands, based on branch/tree rearrangement operations, with each island being the set of all trees of parsimony length $\leq{L}$, connected to each other through a series of included trees that differ by no more than one branch rearrangement.

Maddison (1991) focused on the branch rearrangement operations commonly used in heuristic searches of tree space, nearest-neighbor interchange (NNI), subtree prune-regrafting (SPR), and tree bisection-reconnection (TBR), which are also the bases for corresponding tree-to-tree distances (the minimum numbers of each such operation needed to convert one tree into the other). This facilitates the discovery of islands during tree search without any need for computing tree-to-tree distances and clustering. Otherwise, branch rearrangement operation metric calculations are NP-hard problems (e.g., DasGupta et al. 2000; Allen and Steel 2001; Bordewich and Semple 2005) and, despite attempts to develop efficient algorithms for calculating or approximating branch rearrangement metrics (e.g., Brown and Day 1984; DasGupta et al. 2000; Goloboff 2008; Whidden and Matsen IV 2018), *a posteriori* identification of islands defined by branch rearrangement operation metrics from sets of trees remains computationally expensive. Given that NNI rearrangements are special cases of SPR rearrangements, which are special cases of TBR rearrangements (Bryant 2004; Chernomor et al. 2015), the number of TBR islands will be less than or equal to the number of SPR islands, which in turn will be less than or equal to the number of NNI islands (Maddison 1991).

**Figure 1:**
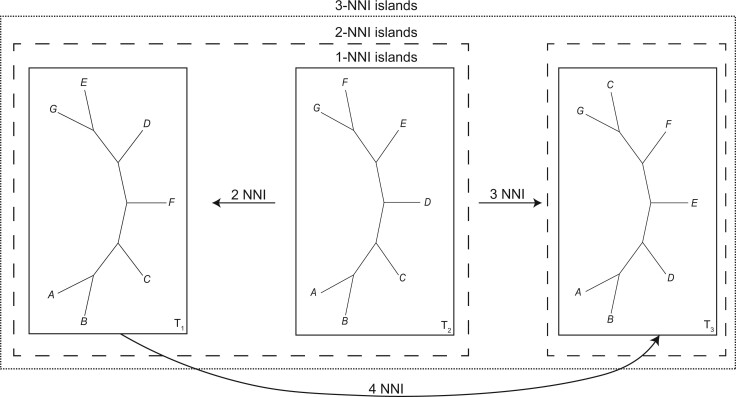
*x*-NNI islands. Three trees separated by more than one nearest neighbor interchange (NNI) branch rearrangement, and thus occupying individual 1-NNI islands (solid line boxes). With the NNI threshold increased to two, $T_{1}$ and $T_{2}$ comprise one 2-NNI island, but $T_{3}$ being more than two NNIs away from $T_{1,2}$ is in its own 2-NNI island (dashed line boxes). When the NNI threshold is three, because all trees are three or fewer NNIs from $T_{2}$, there is a single 3-NNI island comprising all trees (dotted line box). If $T_{2}$ were not present, the NNI threshold required for $T_{1}$ and $T_{3}$ to be in the same *x*-NNI island is four, since that is the NNI distance between $T_{1}$ and $T_{3}$.

We can usefully extend Maddison’s (1991) tree island definition in three ways. Firstly, we allow for islands to be defined using any tree-to-tree distance, not just those based on branch rearrangement operations. This has important consequences for the discovery of islands. Secondly, we define subsets of sufficiently optimal trees (i.e., all trees of length $\leq{L}$ in parsimony, or all trees with likelihood $l$ or better in model-based inference methods), where all adjacent trees differ by less than some threshold distance (such as a maximum of *x* branch rearrangements rather than a single branch rearrangement, or some chosen RF value). This leads us to recognize, 1-NNI islands, which are contained in 2-NNI islands, 3-NNI islands to *x*-NNI islands (Fig. 1), and the same follows for SPR, TBR, and RF islands. In this formulation, it follows that for any set of trees, there will exist some categorization under which the set comprises a single island. Insofar as more or less substantial incongruences between trees may be better reflected by TBR or NNI distances, comparing tree islands defined using different measures and thresholds may help clarify the nature of any incongruence. Thirdly, we can remove the restriction to sufficiently optimal trees and allow islands to be defined for any given set or multiset (i.e., a weighted set) of trees. This is useful for extending the notion of tree islands to the potential multisets (where elements may be repeated) found through Bayesian inference and bootstrap resampling. Lakner et al. (2008) and Höhna et al. (2011) have both employed the notion of islands in a Bayesian context as areas of tree space with a high probability density. In the case of multisets of trees, we use island size to denote the number of distinct tree topologies in an island, island mass to denote the total number of trees in an island, and island density to denote the ratio between island size and mass.

Formally, given (i) a set $\mathcal{T}$ of trees, (ii) a pairwise tree distance function $d : \mathcal{T} \times \mathcal{T} \rightarrow \mathbb{R}_{0}^{+}$, and (iii) a threshold $x \in \mathbb{R}_{0}^{+}$, we define an undirected, edge-weighted graph $G=(V,E)$, where $V=\mathcal{T}$ and there is an edge $(T,T') \in E$ if and only if $d(T,T') \le x$. The *tree islands* of $(\mathcal{T},d,x)$ correspond to connected components of graph $G$. Allowing the tree-to-tree distance function to take on all nonnegative real numbers ($x \in \mathbb{R}_{0}^{+}$) means that this definition encompasses nonbinary trees and can be applied to tree distance metrics that take branch lengths into account.

The definition of islands, irrespective of the tree-to-tree distance used, leads to natural partitionings of a set of trees into mutually exclusive and exhaustive subsets. By contrast, families of trees as defined by Hendy et al. (1988) do not yield mutually exclusive subsets of trees, since a tree may be *x* RF units away from multiple trees ($T_{1}, T_{2}, ... , T_{n} $), and thus belong to multiple *x*-RF families. Note that in the case of binary trees, an NNI of 1 corresponds to an RF of 2 (Chernomor et al. 2015), so that a 2-RF island and a 1-NNI island are equivalent. In contrast, there is no one-to-one correspondence between an SPR (or TBR) and any RF value, so that, for example, from the equations in Chernomor et al. (2015), a 4-RF island might contain trees that are in the same 1-TBR, but not the same 1-SPR islands. Unlike branch rearrangement metric calculations, RF calculations are not an NP-hard problem and RF calculators are widely available. Thus, identifying RF islands *a posteriori* is more tractable than *a posteriori* discovery of islands based on NNIs, SPRs, or TBRs. Of course, other metrics could be used to define and identify islands or other subsets of trees, and what metrics are most helpful under what circumstances remains an open question.

Distinct subsets of trees may provide insights into real biological processes and/or into our attempts to infer relationships, and thus some attention has been paid to the identification of optimal partitions and the associated question of whether a single or multiple consensus trees are required to adequately, or best, represent a set of trees (e.g., Stockham et al. 2002; Bonnard et al. 2006; Guénoche 2013). In this context, interest in islands is justified by their potential to produce natural partitions of tree space without heuristic clustering (or concern for any theoretical “best” clustering). Islands based on branch rearrangement operations are a virtually cost-free byproduct of some searches of tree space, and, as we show, finding islands based on more readily calculated tree-to-tree distances is not intractable. Islands may have a role in investigating what number of consensus trees best summarizes a tree distribution, but our primary practical purpose here is to illustrate the potential negative impact of large island bias and how this may be mitigated.

## EXAMPLE

Pardo et al. (2017) described the fossil amphibian *Chinlestegophis jenkinsi* from the Triassic of North America and sought to infer its relationships to extant and fossil amphibians through Bayesian and parsimony analyses of a data set comprising 76 taxa and 345 morphological characters, both summarized using the majority-rule consensus. Their Bayesian analysis provided high posterior probabilities for a close relationship of *Chinlestegophis* with extant Gymnophiona, and, in contrast to many other studies (e.g., Ruta and Coates 2007; Maddin et al. 2012), only a distant relationship between these and the other living amphibians (Anura and Caudata, collectively Batrachia). This is a surprising and potentially paradigm shifting result with major implications for the meaning, content, age, and evolutionary history of the Lissamphibia (the least inclusive clade including all living amphibians). Congruence between the majority-rule consensus from their parsimony and Bayesian analyses was used to bolster their phylogenetic conclusion.

Pardo et al.’s (2017) parsimony analysis yielded 882 equally optimal trees. Although the majority-rule consensus of these trees is highly congruent with their Bayesian analysis, it is noteworthy that none of the approximately 25 internal branches separating the Gymnophiona from their more traditional placement with Batrachia occur in every MPT and that, in a bootstrap analysis, none garnered support of more than 50%. These observations suggest that the parsimonious interpretation of the data offers little or no support for their novel interpretation of Lissamphibia.

**Figure 2:**
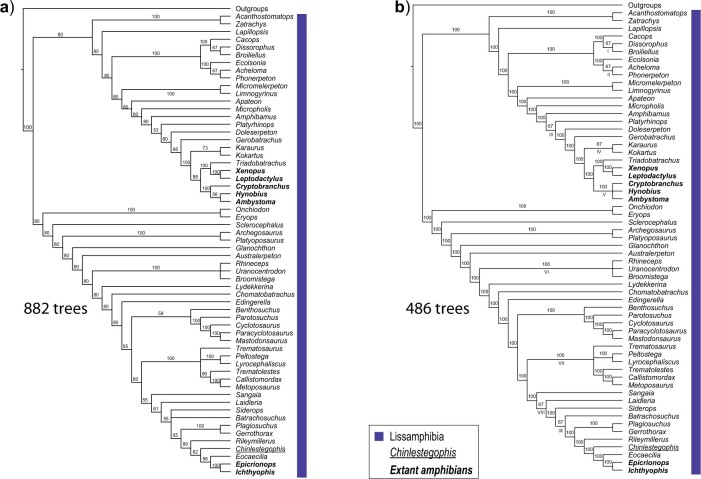
Majority-rule consensus trees of all MPTs from Pardo et al.’s (2017) amphibian data set (a), and of the largest MPT island (b), with the number of MPTs present in each set. All taxa whose placement is the same in all tree islands were collapsed under the label Outgroups. Extant taxa are highlighted in bold, *Chinlestegophis jenkinsi* is underlined and the implied membership of Lissamphibia is highlighted by the shaded box(es). Arabic numerals above branches correspond to branch frequencies and Roman numerals below branches indicate areas of local instability.

Repeating the parsimony analysis, the “Tree-island profiles” in PAUP* v.4a165 (Swofford 2003) reveals that the 882 MPTs are distributed in five 1-TBR islands (that also correspond to five 1-SPR, 1-NNI, and 2-RF islands) and that we refer to simply as islands. The largest island contains more than half of the trees (486), a condition under which we expect the majority-rule consensus of all the trees to be dominated by the largest island. For example, any splits that are common to all the trees in the largest island will necessarily be in the majority-rule consensus of all the MPTs. Thus, in this example, the majority-rule consensus tree of all MPTs (Fig. 2a) and that of the subset of these trees in the largest island (Fig. 2b) share 69 splits, most (64) of which are present in every tree in the largest island, with just one branch in each consensus tree that is unresolved in the other (RF = 2).

The key issue is whether island size can reasonably be taken as a proxy for support. Are inferred relationships in larger islands better supported than those in smaller islands by virtue of the relative sizes of the islands? Sumrall et al. (2001) showed, with examples of bimodal distributions of labile taxa, that subsets of trees may be larger simply because of their having greater local instability. This is not a good reason for preferring relationships in one subset over another, hence Sumrall et al.’s (2001) recommendation that paleontologists should not use the majority-rule consensus to summarize MPTs. As we shall see, the present example illustrates this problem in a multimodal (multiple island) context, demonstrates how Sumrall et al.’s (2001) sensible advice is sometimes ignored or overlooked, and leads to the potential solutions or ameliorations we consider below.

## PARTITIONED-BY-ISLAND CONSENSUS TREES

**Figure 3:**
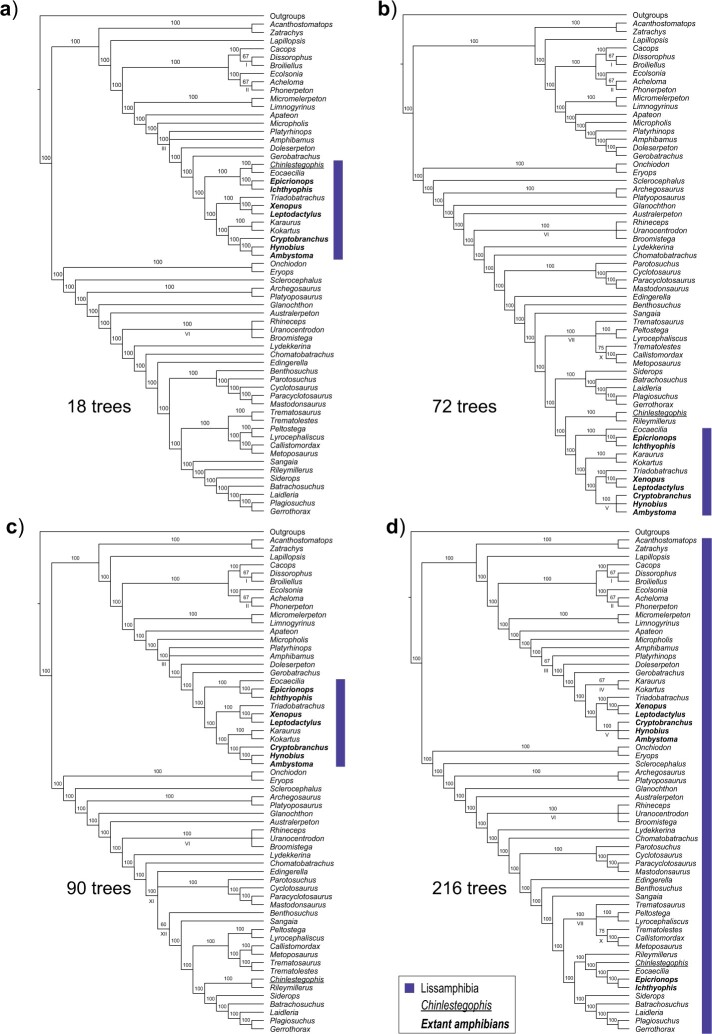
Majority-rule consensus trees for the four smaller tree islands recovered from the parsimony analyses of Pardo et al.’s (2017) data set, with the number of MPTs present in each island (a–d). All taxa whose placement is the same in all tree islands were collapsed under the label Outgroups. Extant taxa are highlighted in bold, *Chinlestegophis jenkinsi* is underlined and the implied members of Lissamphibia is highlighted by the shaded box(es). Arabic numerals above branches correspond to branch frequencies and Roman numerals below branches indicate areas of local instability.

Recognizing that islands may “form sets of trees that might be profitably studied separately" (Maddison 1991, p. 325) and that “[c]hoosing just a single consensus tree may ignore information in the data" (Hendy et al. 1988, p. 358), we can instead generate a consensus of each tree island. If we consider topological variants within islands to be minor, then computing a consensus of each island will help reveal the major variants (Maddison 1991). Applied to our example we obtain one well resolved consensus per island (Figs. 2b and 3a–d). Note that whereas we have used the majority-rule method to produce the partitioned-by-island consensus trees, most of the displayed branches are common to all the relevant input trees and the strict consensus would have been just as useful as it has been in other studies of incongruence (e.g., Soltis and Kuzoff 1995; Hibbett and Donoghue 2001). Our results reveal that a majority, the three smallest of the five islands, feature a more traditional Lissamphibia in which Gymnophiona is closely related to Batrachia. Although an important caveat to their phylogenetic conclusions, Pardo et al. (2017) do not mention that the more traditional Lissamphibia is as parsimonious as the novel relationship inferred using Bayesian inference.

Comparison of the partitioned-by-island consensus trees sheds light on the likely cause of the substantial size disparity between islands. Each consensus shows areas of local instability (indicated either by polytomies or by branches labelled with occurrences of less than 100%). Across the 5 partitioned-by-island consensus trees, there are 12 areas of local instability (all reflecting up to 3 possible alternative resolutions corresponding to NNIs). Of these, nine are present in the largest island, while only four are present in the smallest island, and three occur in every island (Figs. 2b and 3a–d, Table 1). Differences in island size are mostly explained by the number of instances of local instability that the island includes (Pearson’s correlation test: *r* = 0.9053, *P*-value = 0.0345), each of which provides an independent source of alternative relationships. Linear increases in the number of instances of local instability $i$ produce exponential increases (bounded by 3$^i$) in the number of trees as a result of their possible combinations. The link between island size and local instability is confirmed when the bounded maximum size of each island is taken as an alternate measure of instability (*r* = 0.9925, *P*-value = 0.0008). Another line of evidence that links large island size with high local instability, in this data set, is the average degree of each island. If islands result from rearrangments around a small number of poorly resolved nodes then they would be densely connected, with lots of NNI edges connecting trees. We used NetworkAnalyzer (Assenov et al., 2007), available through Cytoscape v.3.7.1 (Shannon et al. 2003), to calculate the average degree of each island, which corresponds to an NNI graph, and found that the smallest and largest islands had, respectively, the lowest (4.33) and highest (9.00) average vertex degrees (Table 1). Furthermore, the highly positive significant correlations between local instability and island sizes were also found between number of local instabilities in and average vertex degree of an island (*r* = 0.9936, *P*-value = 0.0006), and island size and average vertex degree (*r* = 0.9138, *P*-value = 0.0290). Should island size be unrelated to the amount of local instability present in an island, we would not expect this pattern.

**Table 1.** Areas of local instability present in each island

Area	18	72	90	216	486
I	+	+	+	+	+
II	+	+	+	+	+
III	+	$-$	+	+	+
IV	$-$	$-$	$-$	+	+
V	$-$	+	$-$	+	+
VI	+	+	+	+	+
VII	$-$	+	$-$	+	+
VIII	$-$	$-$	$-$	$-$	+
IX	$-$	$-$	$-$	$-$	+
X	$-$	+	$-$	+	$-$
XI	$-$	$-$	+	$-$	$-$
XII	$-$	$-$	+	$- $	$-$
Total	4	6	6	8	9
Average degree	4.33	6.33	6.33	7.67	9.00

Presence of each area of local instability is denoted by a plus sign (+), and the islands are identified by their size. The areas identified by Roman numerals correspond to the areas of local instability labelled in Figures 2b and 3a–d. The average degree for each island (NNI graph) is also provided.

Consider two conflicting relationships, one present in all the trees in a small island and the other present in all trees in a larger island. We contend that confidence in any such branch should be considered independent of the combinatoric effects on island size of regions of instability in other parts of the trees. From that point of view, the effect is a bias toward stable relationships in larger islands.

In this example, the partitioned by island consensus approach also allows us to distinguish between major conflicts reflecting alternative placements of Gymnophiona, Batrachia, and *Chinlestegophis* and more minor patterns of local instability. Among the latter it enables us to distinguish those that are contingent on, and those that are entirely independent (I, II, and VI which are present in all islands) of these major conflicts.

## WEIGHTED-BY-ISLAND-SIZE MAJORITY-RULE CONSENSUS

If we are interested in finding which relationships are supported across multiple islands (and those that are not), island size bias can be avoided by giving all islands equal weight. One means of achieving this is by, under the assumption of island equiprobability, assigning weights inversely proportional to the size of the island to which the input trees belong, so that trees in larger islands will contribute less to the consensus. Here then, trees are assigned a weight of $ {1}/{n_i}$, where $ n_i $ = size of the $ i^{\rm th} $ island. To implement this, tree weights can be added to Nexus format tree files by inserting the expression “[&W $ {1}/{n_i}$]", with $n_i$ replaced by the corresponding island size, before each tree string, and the option “usetreewts" must be set to “yes" in PAUP*. When dealing with multisets, the weight of each unique topology will be ${m_t}/{n_i}$, where $m_t$ corresponds to the number of times a unique topology is present in the tree distribution. As such, in multisets the sum of an island weights might exceed one.

**Figure 4:**
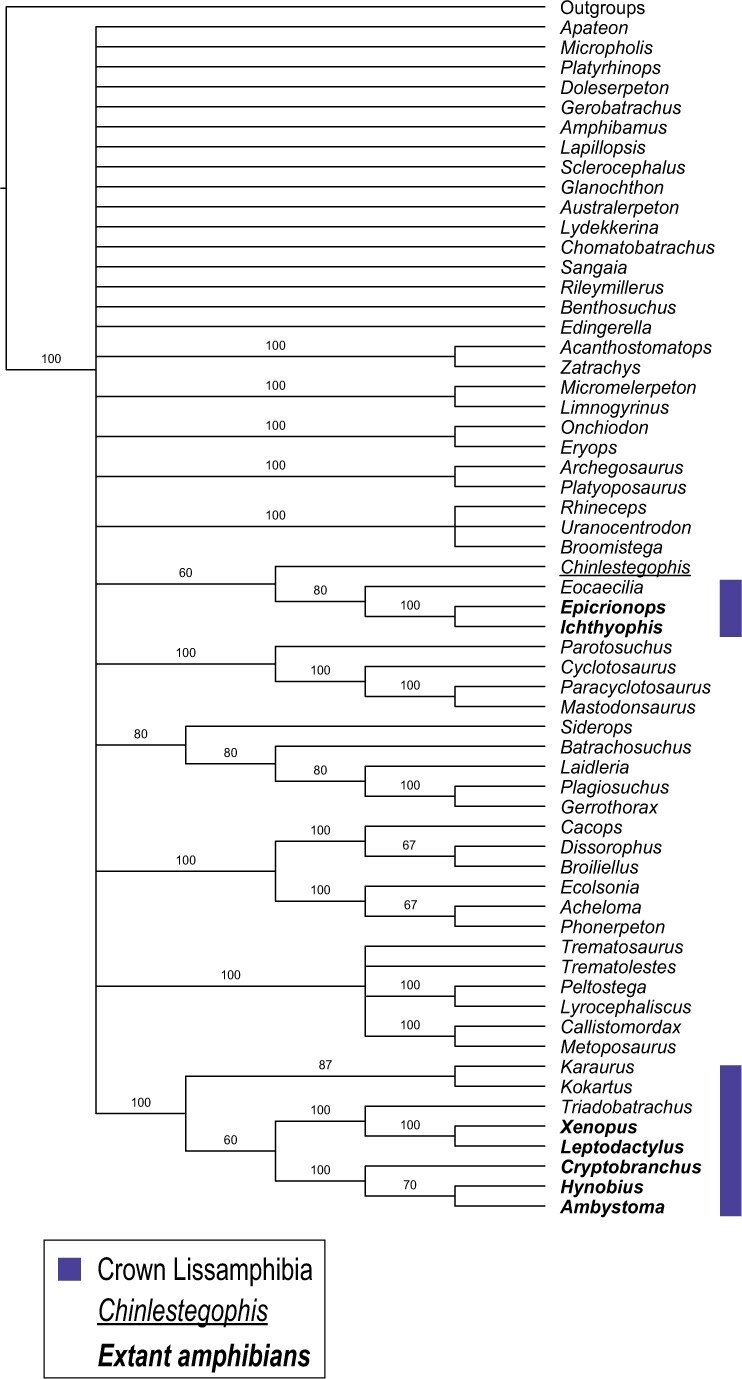
Weighted majority-rule consensus of all MPTs from Pardo et al.’s (2017) amphibian data set. All taxa whose placement is the same in all tree islands were collapsed under the label Outgroups. Extant taxa are highlighted in bold, *Chinlestegophis jenkinsi* is underlined and members of the traditional Lissamphibia crown-group are highlighted by the shaded box(es).

In our example, trees in the smallest island have a weight of ${1}/{18}$, while those in the largest island have a weight of ${1}/{486}$, but each island has a weight of 1. The resulting weighted-by-island-size majority-rule consensus (Fig. 4) is, unsurprisingly, less resolved than each partitioned-by-island consensus. It resembles a strict consensus of all the MPTs with some additional information on splits that occur (with sufficient frequency) in a majority of islands. Interestingly, the weighted-by-island-size consensus recovers a subtree, (*Siderops*, (*Batrachosuchus*,(*Laidleria*,(*Plagiosuchus*, *Gerrothorax*)))), including three splits that are present in all trees of all but the largest island but are not present in the standard majority-rule consensus. To some extent this approach mitigates against failure to acknowledge alternatives that may follow from the uncritical use and unwarranted acceptance of the majority-rule consensus highlighted by Sumrall et al. (2001).

## RAREFIED-BY-ISLAND-SIZE MAJORITY-RULE CONSENSUS

Alternatives to differential weighting of trees are rarefaction and oversampling. One such strategy is to randomly select *n* tree topologies from each island, without replacement, resulting in equal representation of each island in any consensus. The number of trees included per island can be formulated as $n=s\times{p}$, where *s* is the size of the smallest island and *p* the proportion of trees from the smallest island to be included, with $p\in[{1}/{s},1]$. Using $p=1$ will minimize stochastic loss of information from the larger islands, resulting from the random draw of source trees, which may be particularly important if *s* is small (e.g., $\leq{20}$). For multisets, resampled topologies that appear more than once in the tree distribution can be weighted by the number of times they are present in the multiset.

With Pardo et al.’s (2017) MPTs, *s* is 18, so we set $p=1$, giving a total of 90 trees as input to the majority-rule consensus. To further ensure results were not unduly affected by stochastic errors introduced by the random draw of input trees, we repeated the random selection and consensus computations 1000 times (see Appendix for implementation, for single and multiple replicates). Both the strict and standard majority-rule consensus trees of these 1000 rarefied majority-rule consensus have the same topology as the weighted-by-island-size majority-rule consensus (Fig. 4), emphasizing that the two approaches are both attempting to remove large island bias by giving islands equal weight.

## FINDING NNI ISLANDS IN A BAYESIAN TREE DISTRIBUTION

The presence of “tree islands” in Bayesian analyses can affect chain convergence (Lakner et al. 2008; Höhna and Drummond 2011), but, to our knowledge, their effects on summarizing the resulting tree distribution have not been explored, perhaps due to the computational expense of calculating branch rearrangement metrics on typically large samples of often large trees *a posteriori* (DasGupta et al. 2000; Allen and Steel 2001; Bordewich and Semple 2005). We developed a small R (R Core Team, 2019) package that uses the *nni* function in *phangorn* (Schliep, 2011) to iteratively generate the 1-NNI neighborhood of each tree in a distribution and filter the neighborhood to retain only those trees that are also present in the tree distribution. Filtered neighborhoods are then recursively checked for shared trees, if these are present tree neighborhoods are merged until only the 1-NNI islands remain.

Re-analyzing the Pardo et al. (2017) data set in MrBayes v.3.2.6 (Ronquist et al. 2012), under the Mk+I+G model, with two independent runs of four chains for 10 million generations, sampled every 10,000, and a relative burn-in of 25%, yielded a tree distribution containing 1502 unique and equiprobable trees. The majority-rule consensus tree is identical to that reported by Pardo et al. (2017). Applied to this distribution, our R script yielded 1489 1-NNI islands, 1480 comprise a single tree (mass = 1, density = 1), five of the islands contained two trees (mass = 2, density = 1), and four islands were made up of three trees (mass = 3, density = 1), see Supplementary material available on dryad at https://doi.org/10.5068/D14X10 for the tree files. Given that the distribution is composed exclusively of very small islands we can conclude that, unlike in the parsimony analysis, the majority-rule consensus of the Bayesian tree distribution has not been substantially affected by any 1-NNI large island bias.

## MORE ON FINDING ISLANDS *APOSTERIORI*

The discovery of tree islands, both as a general phenomenon and in specific instances, was associated with heuristic tree searches using the branch rearrangement operations (NNI, SPR, and TBR) that are the bases of the tree-to-tree distances used in Maddison’s (1991) original definition of tree islands. Although convenient and helpful for islands to be found as a byproduct of tree searches, the original definition of islands in terms of tree-to-tree distances that are particularly hard to compute *a posteriori* probably has limited subsequent application of the concept of tree islands to investigations of tree distributions more generally. Indeed, to our knowledge, our example above is the first. However, our NNI-island finder R script is very slow, and extended to find 2-NNI islands (see Appendix) it is too slow to be used with anything other than small toy data sets.

However, our revised definition of islands, usefully extends the notion to any tree-to-tree distance, including those whose calculation is not NP-hard, such as RF. Finding islands *a posteriori* based on such metrics is expected to be more efficient and tractable than finding islands based on branch rearrangement metrics. Indeed, given the pairwise distances of a set of trees, finding islands is not difficult (they are the disconnected components of the corresponding graph after removal of edges that are below the threshold value). We have implemented a simple exact algorithm that finds islands from a tree-to-tree distance matrix (see Appendix).

**Figure 5:**
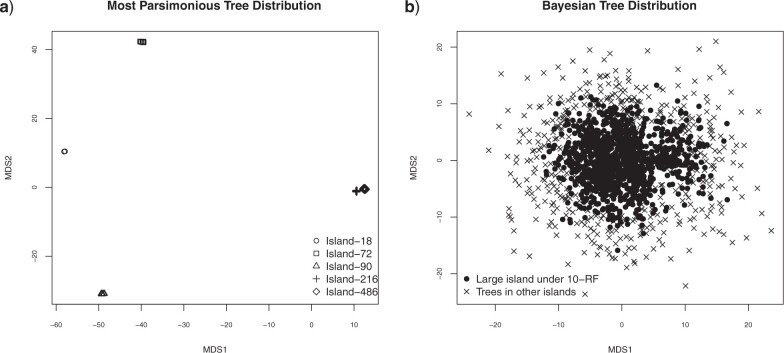
Multidimensional scaling plots of the a) set of MPTs and b) Bayesian posterior distribution of trees obtained from Pardo et al.’s (2017) amphibian data set.

As expected, 2-RF islands found using this algorithm in our example parsimony and Bayesian tree distributions are identical to the corresponding 1-NNI islands. Applying increasingly higher RF thresholds, we found that the island structure of the MPT distribution is robust, with the first change in island structure at $x=12$ (when the two largest islands merged into one). In contrast, the Bayesian island structure is less stable, with a single large island forming at $x=6$ that steadily increases in size with each increment in the RF threshold, while still identifying large numbers of single tree islands and without finding any alternative islands of substantial size (Table 2). This pattern is as expected if we partition a homogenous tree distribution, with increasing thresholds adding more trees to a single island in the center of the distribution that excludes progressively fewer outliers. By $x=10,$ the largest island encompasses over 50% of the trees in the Bayesian tree distribution, and the partitioning of outliers from trees toward the center of the distribution is apparent in a multidimensional scaling based on pairwise RF distances (Fig. 5, see below). At this point, the majority-rule consensus of the single large island is, as expected, identical to that of the full Bayesian tree distribution.

**Table 2.** Number and size of *x*-RF islands found in the Bayesian tree distribution at different RF thresholds

Island size	2-RF	4-RF	6-RF	8-RF	10-RF	12-RF
1	1480	1373	1125	725	397	190
2	5	28	38	22	9	1
3	4	6	5	2	1	0
4	0	2	0	2	0	0
5	0	0	0	2	0	0
6	0	1	0	0	0	0
7	0	1	0	0	0	0
9	0	1	0	0	0	0
25	0	1	0	0	0	0
286	0	0	1	0	0	0
709	0	0	0	1	0	0
1084	0	0	0	0	1	0
1310	0	0	0	0	0	1
Total islands	1489	1413	1169	754	408	192

## OTHER APPROACHES TO PARTITIONING SETS OF TREES

In this section, we briefly consider other methods that have been developed for partitioning sets of trees and, where possible, compare their results with the partitioning by islands of our examples. Of the various clustering approaches that have been proposed few have been compared to tree islands and all are as yet rarely used in practice.

Similar to Hendy et al. (1988) and Höhna and Drummond (2011), we visualized our example empirical tree distributions through multidimensional scaling (MDS). Using the function *metaMDS* in the R package *vegan* v2.5-7 (Oksanen et al. 2020) with the RF distance matrix as input, MDS produces a clear separation of the five islands of MPTs (Fig. 5a) In contrast, MDS produces no partitioning of the Bayesian tree distribution but does reveal that trees in the large 10-RF island are concentrated at the center of the sampled tree space.

Stockham et al. (2002) framed the question of how many consensus trees should be used to summarize a set of trees as a bicriterion problem of complexity (numbers of consensus trees) versus information loss (the distance between the tree distribution being summarized and the tree distribution induced by the strict consensus trees of the proposed clusters). While this remains an important open problem, it motivated and informed their comparison of 1-TBR islands (a byproduct of heuristic parsimony searches) and partitionings of several empirical sets of trees based on two families of clustering algorithms applied to RF distances, *k*-means, and agglomerative clustering (sometimes referred to as linkage clustering). Of the investigated methods they preferred complete linkage clustering. For our MPTs the complete linkage algorithm, computed using the R function *hclust*, yielded five well defined clusters, corresponding to the five islands from our example, and no discernible clusters in the Bayesian tree distribution. Results of the *k*-means analyses, however, change depending on the parameters used, making them harder to interpret. The function *find.clusters*, from the R package *adegenet* v.2.1.3 (Jombart 2008), using the Bayesian Information Criterion (BIC), “goodfit,” and “choose.n.clust=FALSE” settings, identified *k* = 8 as the optimal number of clusters for the MPT distribution and *k* = 10 for the Bayesian distribution. However, if “goodfit” is exchanged for the “diffNgroup” setting, while the optimal number of clusters does not change for the MPT distribution, for the Bayesian distribution the optimal *k* becomes 2.

More recently, Tahiri et al. (2018) modified the *k*-medoids algorithm to work with RF distances and compared its performance under squared and nonsquared RF and the Silhouette and Cali´ski–Harabasz validity indices. Differently from Stockham et al. (2002), they sought to provide an optimal set of majority-rule (rather than strict) consensus trees and they made no comparison to islands. Tahiri et al. (2018) found that their *k*-medoids algorithm performs best with the nonsquared RF distance and the Silhouette validity index, and that it cannot deal with single cluster data sets. We ran their *k*-medoids implementation under the recommended setting, which yielded two clusters for the MPT distribution, corresponding to the extended and restricted Lissamphibia groups of trees, so one cluster includes the three smallest islands, while the other is made up of the two largest islands. For the Bayesian distribution the application accepted the input file but did not generate any output files, suggesting that this corresponds to the *k* = 1 instance, which Tahiri et al.’s (2018) algorithm cannot deal with. Another clustering algorithm is the basis of Guénoche’s (2013) multiple consensus tree method, but we were unable to find an implementation that would allow any investigation of this method.

An alternative to distance-based clustering algorithms are graph-based methods. Bonnard et al. (2006) introduced the multipolar consensus which defines a minimal set of consensus trees (poles) that display all the splits that occur above some minimum frequency ($\alpha$) in the input trees, and implemented a greedy heuristic graph-coloring algorithm based on split compatibility to approximate the multipolar consensus. At $\alpha = 0.5,$ the method yields the majority-rule consensus. Applied to our example data sets, the multipolar consensus, with the default $\alpha = 0$ (i.e., including all splits), identified eight poles from the MPT distribution, four of which are relatively well resolved and are most similar to the partitioned-by-island consensus trees of each of the three smallest and the largest islands, the other four poles are mostly unresolved. For the Bayesian tree distribution, 13 poles were identified, but only one is well resolved. At $\alpha = 0.4$, the multipolar consensus identifies two poles in the MPT distribution, which are most similar to the partitioned consensus trees of each of the two largest islands.

Finally, an approach originally designed to partition sets of gene trees is based on the notion of trees of trees (Nye 2008; Darlu and Guénoche 2011), where each tree topology becomes a leaf in a tree, and the nodes represent intermediate topologies between the input trees. We were unable to find an implementation of Darlu and Guénoche’s (2011) TreeOfTrees and our tree distributions are too large for Nye’s (2008) metaTree (both the web-based and stand-alone implementations). As a rough proxy (in which nodes do not represent intermediate tree topologies) we computed neighbor-joining (NJ) and unweighted pair group method with arithmetic mean (UPGMA) trees from the RF distance matrices (e.g., Graham et al. 1998). These methods also found the five islands of MPTs and no clear partitioning of the Bayesian tree distribution.

While unsurprising that the various methods considered sometimes yield somewhat different partitionings, it is noteworthy that several produce partitionings that are either identical to or otherwise similar to islands.

## DISCUSSION

Historically, most users of parsimony were interested in discovering relationships that are present in every most parsimonious tree, leading to a strong preference for the exclusive use of the strict consensus to summarize most parsimonious trees, and a focus on the unambiguously or strictly supported relationships they display (Nixon and Carpenter 1996). Other relationships might be considered unsupported but there is a difference between a relationship that is present in some MPTs and a relationship that is not present in any MPT. The former seems somewhat better, if ambiguously, supported by the parsimonious interpretation of the data. It is tempting to go further and interpret the frequencies of occurrence of alternative ambiguously supported relationships in a set of MPTs as a measure of their relative support. This would follow from an assumption that, for example, all MPTs are equally probable. However, if there are multiple islands of MPTs, alternative measures of support would follow from an assumption that all islands are equally probable.

Each of the potential assumptions (equiprobable trees or equiprobable islands) might be justified by appeal to the principle of indifference (Keynes 1921). However, both assumptions can be met simultaneously only if all MPTs belong to a single island or all islands have exactly the same size. Otherwise, as our example shows, they can lead to very different and contradictory conclusions. In general, we expect such disagreements to be more likely and profound the greater the asymmetries in island size. Clearly, when confronted with multiple islands of disparate sizes, it is sensible to try to understand the causes of the differences.

In our parsimony example, island size is highly correlated with the number of areas of local instability and is explained by their combinatorial consequences. This argues against island size being correlated with the probability of the island containing the correct tree, against equiprobability of trees, and against the use of the majority-rule consensus, because of the unjustifiable large island bias that ensues. The partitioned-by-island consensus reveals five major variants involving alternative interrelationships of Gymnophiona, Batrachia, and *Chinlestegophis*, and helps in understanding the substantial large island bias due to different combinations of more local instability. In contrast to the majority of trees, the majority of islands recovered the traditional Lissamphibia (Figs. 3a–c). Both the majority of trees and the majority of islands have *Chinlestegophis* closely related to Gymnophiona (Figs. 2b and 3a, b, d). Interestingly, Schoch et al.’s (2020) recent analyses of a data set slightly modified from that of Pardo et al. (2017) recovered the restricted Lissamphibia crown group, with *Chinlestegophis* only distantly related (similar to Fig. 3c), emphasizing that robust inferences of amphibian interrelationships, i.e. inferences that are insensitive to minor variations in the underlying data and/or method of analysis, may be hard to obtain. More detailed exploration of the phylogenetic relationships seemingly supported by the data is facilitated by the variant consensus approaches and may be especially intriguing for groups whose evolutionary history is still being debated, including amphibians (reviewed in Marjanović and Laurin 2019).

Sumrall et al. (2001) recognized the large island bias issue in bimodal tree distributions, warned against using the majority-rule consensus to summarize MPTs, and advocated the sole use of the strict consensus. However, that strict consensus trees can be very poorly resolved has seemingly motivated the use of less strict methods and, over time, Sumrall et al.’s (2001) findings and recommendations have been increasingly overlooked or forgotten. Revisiting the issue, we also urge caution against uncritical use of the majority-rule consensus. If the strict consensus is poorly resolved, then the partitioned-by-island consensus, where islands are summarized individually, can be particularly useful in distinguishing major alternatives and local instabilities. Reduced consensus methods (Wilkinson 1994) may also be helpful in this context. If island sizes are disparate, then simple modifications to the majority-rule consensus, through weighting or rarefaction can remove any large island bias from a unitary consensus summary, if such is needed. Our preference is for exploration and flexible use of multiple consensus methods. Discovery of islands should motivate interest in their biological or methodological significance, and discovery of disparate sizes raises the possibility of large-island bias, and should motivate further assessment of the cause of the size disparity and whether it should impinge on our assumptions of equiprobability of, for example, trees or islands. Note that while we have focused on the majority-rule consensus and the attendant issue of large island bias, researchers may choose to investigate or summarize islands with whichever approaches they prefer, including construction of any form of consensus tree or network.

As we have shown, the notion of islands is extendable to methods that can produce multisets of trees or where the sampled trees are not optimal *per se*, but are due to resampling methods or come from regions of tree space with sufficiently high probability densities. Bayesian and resampling analyses can provide direct evidence that trees are not equiprobable, because each topology can be sampled multiple times, such that island size (number of unique topologies) is less than the sampling of trees from an island (island mass). Whereas island mass should be driven by the posterior probabilities/frequency of sampling of the included trees given the data, island size differences may result in large island biases, which would be of concern. However, the Bayesian distribution from our example revealed no great disparities in 1-NNI/2-RF island sizes, with the number of islands very close to the number of trees. Thus both equiprobable tree and equiprobable island assumptions seem reasonable enough. Furthermore, that increasing the RF threshold only partitions the Bayesian tree distribution into a single large “central” island, and many small islands of outliers is consistent with the tree distribution being homogeneous. Thus, island structure provides no basis for questioning the use of the majority-rule consensus to summarize the results of the Bayesian analysis.

Unfortunately, given that computing branch rearrangement metrics *a posteriori* is an NP-hard problem (DasGupta et al. 2000; Allen and Steel 2001; Bordewich and Semple 2005), finding islands based on these metrics in tree distributions from Bayesian and resampling analyses will be intractable in many cases. This is particularly unfortunate if topological differences that are readily described by such branch rearrangements are potentially linkable to or suggestive of specific evolutionary processes and/or analytical artifacts. For example, we suggest that whereas single NNI moves might represent stochastic error, SPR moves (that are not NNIs) might indicate instances of horizontal gene transfer, and TBR moves (that are not SPRs) might indicate local rooting problems such as might result from long branch attraction.

Our method of finding 1-NNI islands *a posteriori* directly (i.e., without computing NNI distances), is of limited use for large tree distributions, because it replaces distance metric calculations with very large numbers of pairwise comparisons of trees. However, our extended definition of tree islands allows for the use of any tree-to-tree distance metrics to define islands and makes it possible for islands to be identified *a posteriori* and for their causes and consequences to be explored. It reflects our point of view that islands are interesting more for the natural way in which they partition a set of trees than for any specific tree-to-tree distance that they were originally based upon. As such tree islands are complementary to other means of data exploration that involve attempts at partitioning sets of trees in order to provide better summaries and promote better understanding.

Other extensions to the notion of islands might be helpful. Allowing for trees with partially overlapping leaf sets might be achievable through generalized tree-to-tree distances (see Cotton and Wilkinson 2007) and allow clustering of gene trees without having to prune/regraft taxa and might also help shed light on the phenomenon of tree terraces (Sanderson et al. 2011; Sanderson et al. 2015). Another possible extension might be to node-labeled trees, this would be particularly interesting given the recent drive to solve the single versus multiple consensus problem in cancer phylogenetics (Govek et al. 2018; Aguse et al. 2019). Current methods are based exclusively on graph-based clustering, using a variety of distances for rooted trees (Govek et al. 2018; Aguse et al. 2019) that could conceivably be used to define and find islands.

## SOFTWARE AVAILABILITY

R package available on GitHub: https://github.com/ anaserrasilva/islandNeighbours.

## SUPPLEMENTARY MATERIAL

Data available from the Dryad Digital Repository: https://doi.org/10.5068/D14X10

## APPENDIX I

### Implementation of rarefied-by-island-size consensus

The rarefied-by-island-size majority rule consensus was implemented as follows, the randomised tree sets were obtained in R v.3.5.3 (R Core Team, 2019) with the following command, *t(replicate(1000, unlist(list(sort(sample.int(18, 18)), sort(sample.int(72,18) + 18), sort(sample.int(90, 18) + 90), sort(sample.int(216, 18) + 180), sort(sample.int(486, 18) + 396)))))*, where the values following the addition sign correspond to the sum of the length of all islands smaller than the island being sampled. The resulting table was edited by substituting all "ˆ" (start of line) by "\t*contree* ", and all "$\$$" (end of line) by "/ *majrule=yes strict=no treefile=RarefiedM50-#.tre*;\n", the edited text was then inserted into a Nexus file containing the PAUP* (Swofford, 2003) block and the output file numbers edited manually. If a single replicate is desired the R command should be *c(sort(sample.int(18, 18)), sort(sample.int(72,18) + 18), sort(sample.int(90, 18) + 90), sort(sample.int(216, 18) + 180), sort(sample.int(486, 18) + 396))*, and the regular expressions for text manipulation do not need to be modified beyond changing the desired treefile name.

## APPENDIX II

### Description of exact island extraction algorithms

*1. Exhaustive search for *x*-NNI island extraction*

Step 1:

 Given a tree distribution

 For each tree in the distribution:

  Generate 1-NNI neighbourhood of tree:

   For each tree in neighbourhood generate its 1-NNI neighbourhood:

    Repeat $\textit{x}-2$ times

  Filter the *x*-neighbourhood for those trees shared with the tree distribution

Step 2:

 When all filtered neighbourhoods have been identified:

  Compare and merge filtered neighbourhoods with shared trees:

  Recurse until only *x*-NNI islands are left

This algorithm is modified from the one used for the extraction of 1-NNI islands, and requires two functions to be implemented: one to generate the filtered neighbourhoods, and another to compare and merge them.

The 2-NNI island extraction is implemented in our R package. The current exhaustive search implementation is bounded by $(2(n-3))^{x}\times{t}$ non-unique trees, where *n* is the length of the leaf set, $2(n-3)$ is the size of a tree’s 1-NNI neighbourhood, $x=2$ is the *x*-NNI threshold, and *t* is the size of the tree distribution.

*2. Island extraction from a matrix of any pairwise tree-to-tree distances (D).*

Choose a threshold *x*-D

Create a vector with the tree distribution’s length and all values set to property *a*

(Indices in the tree distribution and property vector have 1:1 correspondence)

Set the first instance of *a* to *b*

Find the trees within *x*-D of first tree and change them to property *b* in vector

For all but the first tree:

  Find the trees within *x*-D of trees with property *b*:

   Set corresponding vector indices to *b*

  For all trees in the distribution:

   Find the trees within *x*-D of trees with property *b*:

    Set corresponding vector indices to *b*

Remove all trees with property *b* from tree distribution

Recurse until all *x*-D islands have been extracted

This approach is analogous to graph colouring (properties *a*, *b*). It is implemented in our R package together with the calculation of Robinson-Foulds (RF, Robinson and Foulds 1981) distance matrix.

## References

[B1] Aguse N. , Qi, Y. El-Kebir M. 2019. Summarizing the solution space in tumor phylogeny inference by multiple consensus trees. Bioinformatics 35:i408–i416. 31510657 10.1093/bioinformatics/btz312 PMC6612807

[B2] Allen B.L. , Steel M. 2001. Subtree transfer operations and their induced metrics on evolutionary trees. Ann. Combin. 5:1–15.

[B3] Assenov Y. , RamÃ-rez F., Schelhorn S.-E., Lengauer T., Albrecht M. 2007. Computing topological parameters of biological networks. Bioinformatics 24:282–284. 18006545 10.1093/bioinformatics/btm554

[B4] Bonnard C. , Berry V., Lartillot N. 2006. Multipolar consensus for phylogenetic trees. Syst. Biol. 55:837–843. 17060203 10.1080/10635150600969880

[B5] Bordewich M. , Semple C. 2005. On the computational complexity of the rooted subtree prune and regraft distance. Ann. Comb. 8:409–423.

[B6] Brown E.K. , Day W.H.E. 1984. A computationally efficient approximation to the nearest neighbor interchange metric. J. Classif. 1:93–124.

[B7] Bryant D. 2004. The splits in the neighborhood of a tree. Ann. Comb. 8:1–11.

[B8] Chernomor O. , Minh B.Q., von Haeseler A. 2015. Consequences of common topological rearrangements for partition trees in phylogenomic inference. J. Comput. Biol. 22:1129–1142. 26448206 10.1089/cmb.2015.0146 PMC4663649

[B9] Coiffard C. , Mohr B.A., Bernardes-de Oliveira M.E. 2013. *Jaguariba wiersemana* gen. nov. et sp. nov., an Early Cretaceous member of crown group Nymphaeales (Nymphaeaceae) from northern Gondwana. Taxon 62:141–151.

[B10] Cotton J.A. , Wilkinson M. 2007. Majority-rule supertrees. Syst. Biol. 56:445–52. 17558966 10.1080/10635150701416682

[B11] Darlu P. , Guénoche A. 2011. TreeOfTrees method to evaluate the congruence between gene trees. J. Classif. 28:390–403.

[B12] DasGupta B. , He X., Jiang T., Li M., Tromp J., Zhang L. 2000. On computing the nearest neighbor interchange distance. In: Du D.-Z., Pardalos P. M., Wang J., editors. Discrete mathematical problems with medical applications. DIMACS series in discrete mathematics and theoretical computer science. Providence (RI): American Mathematical Society. p. 125–142.

[B13] Goloboff P.A. 2008. Calculating SPR distances between trees. Cladistics 24:591–597. 10.1111/j.1096-0031.2007.00189.x 34879631

[B14] Govek K. , Sikes C., Oesper L. 2018. A consensus approach to infer tumor evolutionary histories. In: Proceedings of the 2018 ACM International Conference on Bioinformatics, Computational Biology, and Health Informatics. New York (NY): Association for Computing Machinery. p. 63–72.

[B15] Graham S.W. , Kohn J.R., Morton B.R., Eckenwalder J.E., Barrett S.C.H. 1998. Phylogenetic congruence and discordance among one morphological and three molecular data sets from Pontederiaceae. Syst. Biol. 47:545–567. 12066301 10.1080/106351598260572

[B16] Guénoche A. 2013. Multiple consensus trees: a method to separate divergent genes. BMC Bioinf. 14:46. 10.1186/1471-2105-14-46 PMC3599424 23394478

[B17] Gunnell G.F. , Smith R., Smith T. 2017. 33 million year old *Myotis* (Chiroptera, Vespertilionidae) and the rapid global radiation of modern bats. PLoS One 12:e0172621. 28273112 10.1371/journal.pone.0172621 PMC5342209

[B18] Hendy M.D. , Little C.H.C., Penny D. 1984. Comparing trees with pendant vertices labelled. SIAM J. Appl. Math. 44:1054–1065.

[B19] Hendy M.D. , Steel M.A., Penny D., Henderson I.M. 1988. Families of trees and consensus. In: Block H. H., editor. Classification and related methods of data analysis. New York: Elsevier. p. 355–362.

[B20] Hibbett D.S. , Donoghue M.J. 2001. Analysis of character correlations among wood decay mechanisms, mating systems, and substrate ranges in homobasidiomycetes. Syst. Biol. 50:215–242. 12116929

[B21] Höhna S. , Drummond A.J. 2011. Guided tree topology proposals for Bayesian phylogenetic inference. Syst. Biol. 61:1–11. 21828081 10.1093/sysbio/syr074

[B22] Jombart T. 2008. adegenet: a R package for the multivariate analysis of genetic markers. Bioinformatics 24:1403–1405. 18397895 10.1093/bioinformatics/btn129

[B23] Keynes J. 1921. A treatise on probability. London: Macmillan and Co., Limited.

[B24] Lakner C. , van der Mark P., Huelsenbeck J.P., Larget B., Ronquist F. 2008. Efficiency of Markov chain Monte Carlo tree proposals in Bayesian phylogenetics. Syst. Biol. 57:86–103. 18278678 10.1080/10635150801886156

[B25] Maddin H.C. , Jenkins Jr F.A., Anderson J.S. 2012. The braincase of *Eocaecilia micropodia* (Lissamphibia, Gymnophiona) and the origin of caecilians. PLoS One 7:e50743. 23227204 10.1371/journal.pone.0050743 PMC3515621

[B26] Maddison D.R. 1991. The discovery and importance of multiple islands of most-parsimonious trees. Syst. Biol. 40:315–328.

[B27] Margush T. , McMorris F. 1981. Consensus n-trees. Bull. Math. Biol. 43:239–244.

[B28] Marjanović D. , Laurin M. 2019. Phylogeny of Paleozoic limbed vertebrates reassessed through revision and expansion of the largest published relevant data matrix. PeerJ 6:e5565. 30631641 10.7717/peerj.5565 PMC6322490

[B29] Nixon K.C. , Carpenter J.M. 1996. On consensus, collapsibility, and clade concordance. Cladistics 12:305–321. 10.1111/j.1096-0031.1996.tb00017.x 34920619

[B30] Nye T.M. 2008. Trees of trees: an approach to comparing multiple alternative phylogenies. Syst. Biol. 57:785–794. 18853364 10.1080/10635150802424072

[B31] Oksanen J. , Blanchet F.G., Friendly M., Kindt R., Legendre P., McGlinn D., Minchin P.R., O’Hara R.B., Simpson G.L., Solymos P., Stevens M.H.H., Szoecs E., Wagner H. 2020. vegan: community ecology package. R package version 2.5-7. Available from: https://CRAN.R-project.org/package=vegan.

[B32] Olmstead R.G. , Bremer B., Scott K.M., Palmer J.D. 1993. A parsimony analysis of the Asteridae sensu lato based on rbcL sequences. Ann. Mo. Bot. Gard. 800:700–722.

[B33] Olmstead R.G. , Palmer J.D. 1994. Chloroplast DNA systematics: a review of methods and data analysis. Am. J. Bot. 81:1205–1224.

[B34] Pardo J.D. , Small B.J., Huttenlocker A.K. 2017. Stem caecilian from the Triassic of Colorado sheds light on the origins of Lissamphibia. Proc. Natl. Acad. Sci. USA 114:E5389–E5395. 28630337 10.1073/pnas.1706752114 PMC5502650

[B35] R Core Team. 2019. R: A language and environment for statistical computing. R Foundation for Statistical Computing Vienna, Austria.

[B36] Robinson, D. F. and Foulds L. R.. 1981. Comparison of phylogenetic trees. Math. Biosci. 53:131–147.

[B37] Ronquist F. , Teslenko M., van der Mark P., Ayres D.L., Darling A., Höhna S., Larget B., Liu L., Suchard M.A., Huelsenbeck J.P. 2012. Mrbayes 3.2: efficient Bayesian phylogenetic inference and model choice across a large model space. Syst. Biol. 61:539–542. 22357727 10.1093/sysbio/sys029 PMC3329765

[B38] Ruta M. , Coates M.I. 2007. Dates, nodes and character conflict: addressing the lissamphibian origin problem. J. Syst. Paleontol. 5:69–122.

[B39] Sanderson M.J. , McMahon M.M., Stamatakis A., Zwickl D.J., Steel M. 2015. Impacts of terraces on phylogenetic inference. Syst. Biol. 64:709–726. 25999395 10.1093/sysbio/syv024

[B40] Sanderson M.J. , McMahon M.M., Steel M. 2011. Terraces in phylogenetic tree space. Science 333:448–450. 21680810 10.1126/science.1206357

[B41] Schliep K.P. 2011. phangorn: phylogenetic analysis in R. Bioinformatics 27:592–593. 21169378 10.1093/bioinformatics/btq706 PMC3035803

[B42] Schoch R.R. , Werneburg R., Voigt S. 2020. A Triassic stem-salamander from Kyrgyzstan and the origin of salamanders. Proc. Natl. Acad. Sci. USA 117:11584–11588. 32393623 10.1073/pnas.2001424117 PMC7261083

[B43] Shannon P. , Markiel A., Ozier O., Baliga N.S., Wang J.T., Ramage D., Amin N., Schwikowski B., Ideker T. 2003. Cytoscape: a software environment for integrated models of biomolecular interaction networks. Genome Res. 13:2498–2504. 14597658 10.1101/gr.1239303 PMC403769

[B44] Sharkey M.J. , Leathers J.W. 2001. Majority does not rule: the trouble with majority-rule consensus trees. Cladistics 17:282–284. 10.1111/j.1096-0031.2001.tb00124.x 34911242

[B45] Sharkey M.J. , Stoelb S., Miranda-Esquivel D.R., Sharanowski B.J. 2013. Weighted compromise trees: a method to summarize competing phylogenetic hypotheses. Cladistics 29:309–314. 10.1111/cla.12000 34809409

[B46] Sokal R.R. , Rohlf F.J. 1981. Taxonomic congruence in the Leptopodomorpha re-examined. Syst. Zool. 30:309–325.

[B47] Soltis D.E. , Kuzoff R.K. 1995. Discordance between nuclear and chloroplast phylogenies in the *Heuchera* group (Saxifragaceae). Evolution 49:727–742. 28565145 10.1111/j.1558-5646.1995.tb02309.x

[B48] Stockham C. , Wang L.-S., Warnow T. 2002. Statistically based postprocessing of phylogenetic analysis by clustering. Bioinformatics 18:S285–S293. 12169558 10.1093/bioinformatics/18.suppl_1.s285

[B49] Sumrall C.D. , Brochu C.A., Merck J.W. 2001. Global lability, regional resolution, and majority-rule consensus bias. Paleobiology 27:254–261.

[B50] Swofford, D. L. 2003. Paup*: phylogenetic analysis using parsimony (*and other methods). version 4.0 a165. Sinauer Associates, Sunderland, Massachusetts.

[B51] Tahiri N. , Willems M., Makarenkov V. 2018. A new fast method for inferring multiple consensus trees using k-medoids. BMC Evol. Biol. 18:48. 29621975 10.1186/s12862-018-1163-8 PMC5887197

[B52] Taylor D.W. , Brenner G.J., Basha S.H. 2008. *Scutifolium jordanicum* gen. et sp. nov.(Cabombaceae), an aquatic fossil plant from the Lower Cretaceous of Jordan, and the relationships of related leaf fossils to living genera. Am. J. Bot. 95:340–352. 21632359 10.3732/ajb.95.3.340

[B53] Whidden C. , Matsen IV F.A. 2018. Efficiently inferring pairwise subtree prune-and-regraft adjacencies between phylogenetic trees. In: Nebel M., Wagner S., editors. 2018 Proceedings of the Fifteenth Workshop on Analytic Algorithmics and Combinatorics (ANALCO). Philadelphia (PA): Society for Industrial and Applied Mathematics. p. 77–91.

[B54] Wilkinson M. 1994. Common cladistic information and its consensus representation: reduced Adams and reduced cladistic consensus trees and profiles. Syst. Biol. 43:343–368.

[B55] Wilkinson M. , Benton M.J. 1996. Sphenodontid phylogeny and the problems of multiple trees. Philos. Trans. R. Soc. B 351:1–16.

